# Terahertz Mie scattering in tissue: diffuse polarimetric imaging and Monte Carlo validation in highly attenuating media models

**DOI:** 10.1117/1.JBO.30.6.066001

**Published:** 2025-06-04

**Authors:** Erica Heller, Kuangyi Xu, Zachery B. Harris, M. Hassan Arbab

**Affiliations:** Stony Brook University, Department of Biomedical Engineering, Stony Brook, New York, United States

**Keywords:** terahertz time-domain spectroscopy and polarimetry, terahertz time-domain spectroscopic imaging, Mie scattering, tumor budding, degree of polarization, diffused scattering

## Abstract

**Significance:**

Changes in the structure of tissue occur in many disease processes, such as the boundaries of cancerous tumors and burn injuries. Spectroscopic and polarimetric alterations of terahertz light caused by Mie scattering patterns have the potential to be a diagnostic marker.

**Aim:**

We present an analysis of Monte Carlo simulation of Mie scattering of polarized terahertz light from cancerous tumor budding, compare the simulation with experimental results obtained in phantom models, and present an analysis of a polarization-sensitive terahertz scan of an *ex vivo* porcine burn injury.

**Approach:**

Using a Monte Carlo simulation, we modeled the changes in diffuse intensity and degree of polarization of broadband off-specular terahertz light due to scattering particles in highly attenuating tissue. We extracted the Mueller matrix of the tissue using this model and analyzed the Lu-Chipman product decomposition matrices. We compared this model with experimental data from four phantoms consisting of polypropylene particles of varying sizes embedded in gelatin. Finally, we induced a full-thickness burn injury in *ex vivo* porcine skin samples and compared experimental data captured over burned and healthy regions of the tissue.

**Results:**

Simulation revealed contrast in the Stokes vectors and Mueller Matrix elements for varying scattering particle sizes. Experimental phantom results showed contrast between different sizes of scattering particles in degree of polarization and diffuse intensity in agreement with Monte Carlo simulation results. Finally, we demonstrated a similar diffused imaging signal contrast between burned and healthy regions of *ex vivo* porcine skin.

**Conclusion:**

Polarimetric terahertz imaging has the potential to detect structural changes due to biological disease processes.

## 1 Introduction

With the rapid advancement of terahertz (THz) spectroscopic methods, this range of electromagnetic frequencies has attracted more attention in the biomedical optics field in recent years.^1^^,^^2^ Several biological applications of THz imaging have been explored, including *ex vivo* experiments on cancer diagnostics^3^^–^^6^ and *in vivo* studies on skin burn classification.^7^^–^^9^ Typically, these studies have explored the dielectric permittivity of the tissue to examine sample properties and to differentiate between healthy and diseased specimens. Particularly, most THz *in vivo* applications focus on the water content of different tissue types.^10^^–^^13^ In comparison, very few studies have used the scattering of the THz light from biological tissue as a diagnostic modality. With an increase in signal-to-noise ratio (SNR) and advancements in THz technology, scattering measurements could play an important role in the characterization and classification of some tissue types.

The study of the dielectric permittivity of tissue often assumes a homogeneous structure or alternatively employs an effective medium theory.^14^^–^^16^ The high absorption of terahertz waves by water content of the tissue has, therefore, remained as the main source of diagnostic signal contrast mechanism. However, this approach can be oversimplifying in many disease conditions. In particular, at the edges of certain types of cancerous tumors, such as colon cancer, oral squamous cell carcinoma, and breast cancer, the formation of tumor budding and poorly differentiated clusters can occur, where parts of the tumor break off and form small clusters within otherwise healthy tissue.^17^ It has been shown that these features can be independent prognostic factors for lymph node metastasis and patient survival.^18^^,^^19^ Current methods for determining the extent of growth of these features include hematoxylin and eosin (H&E) staining and immunohistochemistry.^20^ Other disease conditions can also create a change in the scattering properties of tissue. For example, burns can cause the destruction of higher-scattering features such as hair follicles and sweat glands in the skin.^21^^,^^22^

A promising method for studying these tissue media is through the detection of polarimetric signal contrast in scattered light.^23^^,^^24^ Xu and Arbab recently developed a model for Mie scattering of broadband terahertz light in biological media using Monte Carlo methods and validated the computational results against analytical solutions.^25^ Light-scattering models have been used extensively in biophotonics to evaluate the potential for various modalities of optical imaging to detect different forms of the skin,^26^ gastric,^27^ breast,^28^^,^^29^ prostate,^30^ colon,^31^^,^^32^ and cervical cancer subtypes^33^ and to investigate contrast mechanisms and sensitivity to tissue changes with disease.^34^ In addition, polarized scattering light spectroscopy has been investigated for use in endoscopic^35^ and laparoscopic imaging of various gastrointestinal diseases^36^ and cancers, including colonic polyps^37^ and peritoneal lesions.^38^ Similarly, polarization measurements of the reflected THz light have been explored for delineation between cancerous and healthy tissue;^39^^,^^40^ however, the mechanisms behind the polarization response differences between the types of tissue are yet to be studied in THz frequencies. Recently, Lopushenko et al. proposed a generalized Monte Carlo model for tracking the evolution of the polarization of light in turbid media using a combined Stokes and Jones-Mueller formalism. However, the application of this model is limited to low absorption media, whereas biological tissues generally exhibit high attenuation in the THz regime.^41^ Given the recent development of various THz ellipsometry and polarimetry systems,^42^^–^^47^ there is a timely need for computational models that can explain the observed phenomena.

In this paper, we present Monte Carlo simulation and experimental phantom as well as *ex vivo* measurements of polarization changes due to Mie scattering from structures within the tissue, indicating different degrees of disease progression. We explore the potential utility of diffuse scattering measurements for the THz polarimetric diagnosis of disease processes and other THz biophotonics applications.

## 2 Methods

### 2.1 Terahertz Diffused Scattering Spectral Measurement

**Fig. 1:**
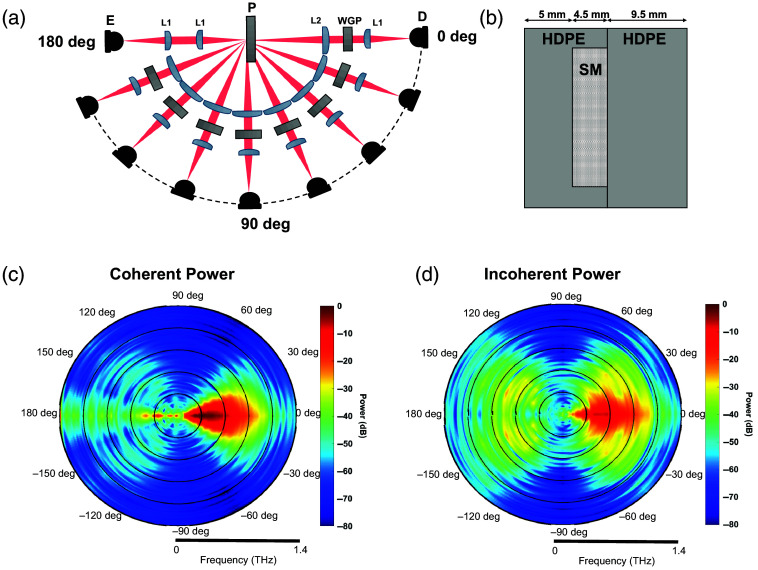
(a) Diagram of the experimental setup. E = emitter, D = detector, L1 = TPX lens with 50-mm focal length, L2 = PTFE lens with 100-mm focal length, WGP = wire-grid polarizer, and P = phantom. (b) HDPE container used for holding scattering material (SM). (c)–(d) Angular distribution of coherent and incoherent power measured from LDPE scattering material. Measurements from 160 deg to 180 deg were made with the addition of a beam-splitter in the beam path.

Broadband terahertz time-domain spectroscopic (THz-TDS) measurements were performed using the experimental setup shown in Fig. 1(a). In the emission arm, a photoconductive antenna (PCA) was excited using a 1560-nm femtosecond laser, which was then focused onto the sample using a pair of collimating and focusing lenses (L1). In the detection arm, which had the ability to rotate from 0 deg to 160 deg, the light passes through a wire-grid polarizer (WGP) between the collimating (L2) and focusing lenses (L1) before it reaches the PCA detector. To quantify the diffused component of the THz scattered light, we used a sample composed of 180-micron low-density polyethylene (LDPE) scattering particles within a high-density polyethylene (HDPE) container with a volume density of $0.3\;g/{\mathrm{cm}}^{3}$ [Fig. 1(b)].^48^ We obtained THz-TDS scattering measurements at 10-deg intervals from 0 deg to 160 deg. Separately, we captured a back-scattering measurement at the 180-deg direction by placing a beam splitter in the emission path and scaling the measurement according to the loss of power through the beam splitter. At each angle, scattering measurements were collected at 36 disjointed locations on the sample container, each 5 mm apart. Each scattering realization included averaging 100 time-domain traces at each sample location. To calculate the coherent and incoherent components of the scattered THz power, the following definitions were used: the average power is given by, $\left\langle I\rangle \propto \langle |E_{s}{|}^{2}\right\rangle$, where the angle brackets represent averaging over the 36 spatially disjoint measurements and the scattered electric field, $E_{s}$, is given in terms of its coherent and incoherent components by $E_{s}=E_{\mathrm{coh}}+E_{\mathrm{incoh}}$. The coherent components of the electric field and power are defined as $E_{\mathrm{coh}}=\langle E_{s}\rangle$ and $I_{\mathrm{coh}}\propto |\langle E_{s}\rangle {|}^{2}$, respectively. The previous definitions give rise to the following equation for the calculation of the incoherent power $I_{\mathrm{incoh}}$

$$\langle E_{s}\cdot E_{s}^{*}\rangle =\langle (E_{\mathrm{coh}}+E_{\mathrm{incoh}})\cdot (E_{\mathrm{coh}}+E_{\mathrm{incoh}}{)}^{*}\rangle =\langle E_{\mathrm{coh}}\cdot E_{\mathrm{coh}}^{*}\rangle +\langle E_{\mathrm{incoh}}\cdot E_{\mathrm{incoh}}^{*}\rangle =I_{\mathrm{coh}}+I_{\mathrm{incoh}}.$$ (1)

Figures 1(c) and 1(d) show the angular distribution of the detected coherent and incoherent power, respectively, from 0.2 to 1.4 THz, normalized by the peak value of the coherent power. We observed a sharp decrease in coherent power from 0 deg to 20 deg, with an increase past 160 deg. In contrast, the incoherent power remained above our system’s detectable threshold (≥60  dB) at all angles except 70 deg to 120 deg. These results show that the diffusely back-scattered beam at about 140 deg is dominated by the incoherent scattered photons that can carry the information regarding tissue structures. Due to this sharp contrast between the incoherent and coherent power in the diffuse backscattering direction, we chose to obtain the subsequent phantom measurements at 140 deg. These scattering data were measured by raster scanning the phantom sample over the beam focus, moving a distance of 2 mm for each pixel, for x- and y-polarization measurements. Two raster scans were performed for each sample, with the polarization state of the WGP and THz detector rotated between each scan. A total of 100 measurements were recorded for each polarization state over a 2 cm by 2 cm area.

### 2.2 Fabrication of Tissue Phantoms with Scattering Particles

Our objective is to emulate a tissue sample in which spherical structures having refractive index and absorption coefficient lower than those of the medium are embedded. Examples of such structures include hair follicles,^49^ empty sweat glands,^50^ or certain types of cancer such as oral squamous cell carcinoma.^51^^,^^52^ We constructed such phantoms using polypropylene particles embedded in gelatin, a commonly used base for tissue phantoms in the THz regime.^53^^–^^56^ A 15% bovine skin gelatin solution (Sigma Aldrich, Inc.) was heated to 40°C. Once the desired temperature was reached, polypropylene particles (MicroPowders, Inc.) were rapidly mixed into the solution. The solution was then poured into a Petri dish to a height of about 5 mm. Because of the rapid viscosity change as the gelatin cools, the particles remained suspended within the gelatin and did not settle. This process was repeated for four phantoms, with different particle sizes and concentrations. However, due to the clumping of the particles during the mixing process, causing the phantoms to differ from the intended parameters, the size and concentrations of the particles were later analyzed using optical microscopy and ImageJ software.^57^

**Fig. 2:**
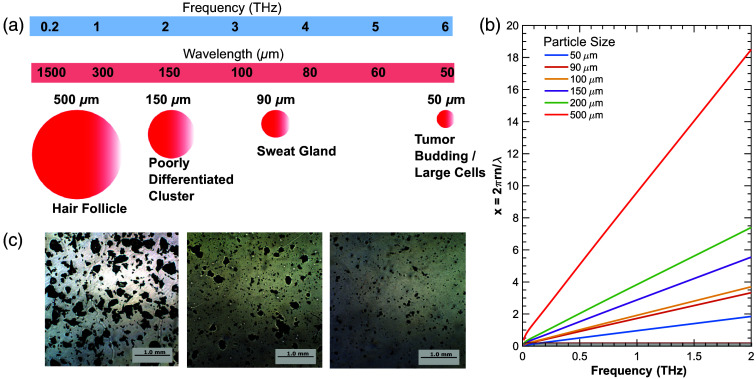
(a) Artistic representation of relevant spherical tissue structure sizes compared to terahertz wavelengths.^58^ (b) Mie scattering parameter for particle sizes 50 to 500  μm. (c) Representative optical microscopy images of fabricated phantoms.

Particle sizes for the phantoms were chosen based on the wavelengths of THz light and the size of relevant tissue structures, such as tumor buds and hair follicles. Figure 2(a) shows a diagram of the sizes of relevant tissue structures compared to the THz wavelengths.^49^^,^^50^^,^^58^ To ensure that the resultant electromagnetic scattering is within the Mie regime, we calculated the size parameter x=2πrn/λ where r is the radius of the particles, n is the refractive index of the host medium (assumed to be the refractive index of the skin), and lambda is the wavelength for relevant particle sizes. As shown in Fig. 2(b), all relevant particle sizes fall within a size parameter between 0.2 and 20 within our system’s usable bandwidth of 0.2 to 1.5 THz, indicating that the primary scattering will be due to Mie theory. The particle sizes for the phantoms were chosen based on the sizes of sweat glands and poorly differentiated tumor clusters that could reasonably be detected with our experimental setup. Figure 2(c) shows examples of three of our phantoms, with varying particle sizes.

**Fig. 3:**
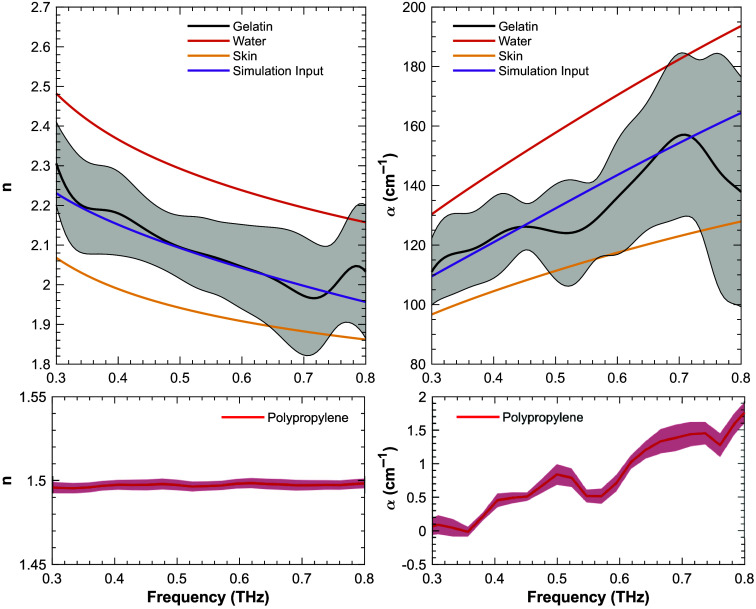
Measured refractive indexes and absorption coefficients of gelatin and polypropylene. Refractive indexes and absorption coefficients of water and skin are included for comparison. Refractive index and absorption coefficient values between water and skin were adopted as our simulation input.

We measured the dielectric properties of the gelatin and polypropylene particles for use in our Monte Carlo model. In the case of the polypropylene particles, we took transmission measurements at the 0-deg angle of our setup through a pellet constructed by pressing the polypropylene particles under a 3000 psi load for ∼1  h. For the gelatin medium, the recently developed PHASR scanner^44^^,^^59^ was used to obtain reflection measurements at normal incidence angle on a pure 15% gelatin sample. The calculated index of refraction and absorption coefficients were averaged across 100 pixels for polypropylene and a representative ROI of 16 pixels for gelatin, shown in Fig. 3. The measured refractive index of the polypropylene particles is slightly lower than the expected value of ∼1.51 in the THz frequency range;^60^^,^^61^ however, this could be due to manufacturing variability or remaining air pockets within the pressed pellet sample. The measured absorption coefficient had a value of $<2\;{\mathrm{cm}}^{-1}$ for the entire bandwidth of our system. The measured refractive index and the absorption coefficient of gelatin were higher than those of the skin,^62^^,^^63^ but below those of water.^14^ For use in our Monte Carlo model, we adopted the values of the refractive index and the absorption coefficient between water and the skin, shown in Fig. 3.

### 2.3 Monte Carlo Simulations

**Fig. 4:**
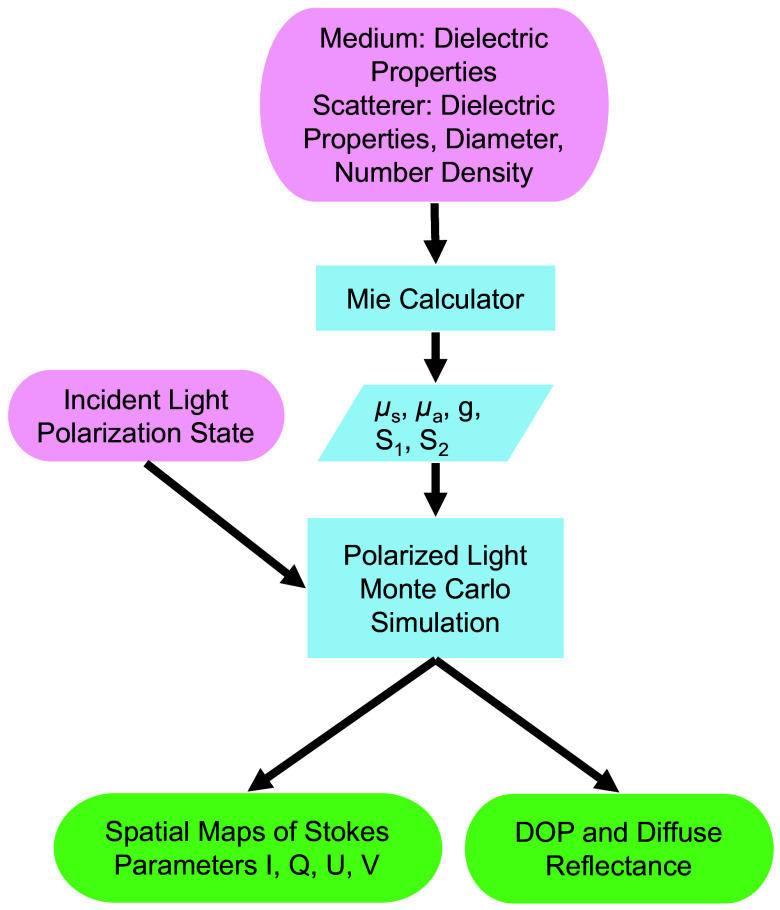
Flowchart indicating the simulation input parameters and the interface between the Mie Calculator and Polarized Light Monte Carlo model.

The multilayer diffused reflectance and polarization Monte Carlo code used for modeling the samples was developed by Xu and Arbab,^25^ based on the Meridian Plane Polarized Light Monte Carlo Code by Ramella-Roman et al.,^64^ with alterations to the Mie scattering parameters to account for an absorbing host medium. Figure 4 describes the flow chart of the altered Polarized Light Monte Carlo code with the addition of a Mie calculator to generate the input parameters. The Mie calculator takes an input of the dielectric properties of the medium and particles, the size of the particles, and the number density of the particles and produces the asymmetric factor g (the average cosine of the scattering angle) and the scattering and absorption coefficients $\mu_{s}$ and $\mu_{a}$, given by^65^

$$\mu_{s}=\frac{2\pi \rho }{|n_{m}{|}^{2}k_{0}^{2}}\overset{\infty }{\underset{j=1}{\sum }}(2j+1)(|a_{j}{|}^{2}+|b_{j}{|}^{2}),$$ (2)

$$\mu_{a}=\frac{2\pi \rho }{R(n_{m})k_{0}^{2}}I[(\overset{\infty }{\underset{j=1}{\sum }}(2j+1)(|c_{j}{|}^{2}\psi_{j}(z){\psi_{j}^{\prime }}^{*}(z)-|d_{j}{|}^{2}\psi_{j}^{\prime }(z)\psi_{j}^{*}(z)))/n_{p}],$$ (3)

where $n_{m}$ and $n_{p}$ are the complex refractive indexes of the medium and particles, respectively, $k_{0}=2\pi /\lambda$ is the wave vector, ρ is the number density of the particles $a_{j}$, $b_{j}$, $c_{j}$, and $d_{j}$ are the Mie coefficients, and $\psi_{j}(z)$ is a Ricatti-Bessel function where $z=n_{p}k_{0}r$. In addition to these parameters, the Mie calculator also produces the scattering matrix that represents the relationship between the incident ($E_{i}$) and scattered ($E_{s}$) field amplitudes using Jones’ calculus

$$[\begin{array}{c} E_{s}^{⊥}\\ E_{s}^{\left|\right|} \end{array}]\propto [\begin{array}{cc} S_{1} & 0\\ 0 & S_{2} \end{array}][\begin{array}{c} E_{i}^{⊥}\\ E_{i}^{\left|\right|} \end{array}].$$ (4)

The Mueller calculus transform of Eq. (4) is given by

$$[\begin{array}{c} I_{s}\\ Q_{s}\\ U_{s}\\ V_{s} \end{array}]\propto [\begin{array}{cccc} S_{11} & S_{12} & 0 & 0\\ S_{12} & S_{11} & 0 & 0\\ 0 & 0 & S_{33} & S_{34}\\ 0 & 0 & -S_{34} & S_{33} \end{array}][\begin{array}{c} I_{i}\\ Q_{i}\\ U_{i}\\ V_{i} \end{array}],$$ (5)

where I, Q, U, and V are the Stokes parameters of the incident and scattered light. The Polarized Light Monte Carlo code produces the spatial Stokes Parameters for a given set of outputs from the Mie Calculator and an initial incident light polarization. Using four different incident light polarizations (specifically horizontal linearly polarized, vertical linearly polarized, 45 deg linearly polarized, and right circularly polarized), the spatial Mueller Matrix parameters can be calculated. In addition, using spatially averaged Stokes parameters from a linearly polarized incident light, the degree of polarization (DOP) of the scattered beam can be calculated as

$$\mathrm{DOP}=\frac{\sqrt{Q^{2}+U^{2}+V^{2}}}{I}.$$ (6)

## 3 Results

### 3.1 Evaluation of Average Particle Size and Density in Phantoms

The particle sizes and concentrations for each phantom were evaluated using optical microscopy and an ImageJ workflow. A threshold was set in ImageJ to discard any detected particles below a diameter of 75  μm, to eliminate any image artifacts and small particles that were unlikely to contribute to the signal. Twenty-five microscope images such as those in Fig. 2(c) were taken of each phantom, and 10 ROIs of a 1×1  mm field of view were delineated for each image, giving a total of 250 measurements of average particle size and concentration for each phantom. These measurements were averaged to give an average particle size and concentration for each phantom as inputs for the simulation. The concentration calculated using this procedure provides the surface density in units of $\text{particles}/{\mathrm{mm}}^{2}$. To convert this quantity to volume density, it was divided by the summation of the depth of field of the microscope and twice the particle diameter size. Due to inhomogeneities within the phantoms, such as clumping of the particles, some phantoms produced inconsistent density and particle size numbers and were therefore excluded from further analysis. Table 1 summarizes the average particle sizes and volume density for the final four phantoms as input parameters for Monte Carlo analysis in the following section.

**Table 1:** Average particle size and volume density of the phantoms.

Phantom	Particle diameter (μm)	Surface density (${\mathrm{particles}/\mathrm{mm}}^{2}$)	Volume density (${\mathrm{mm}}^{-3}$)
A	115	3	10
B	130	2	7
C	180	3.5	8.5
D	280	6.5	10.5

### 3.2 Monte Carlo Simulation Results

**Fig. 5:**
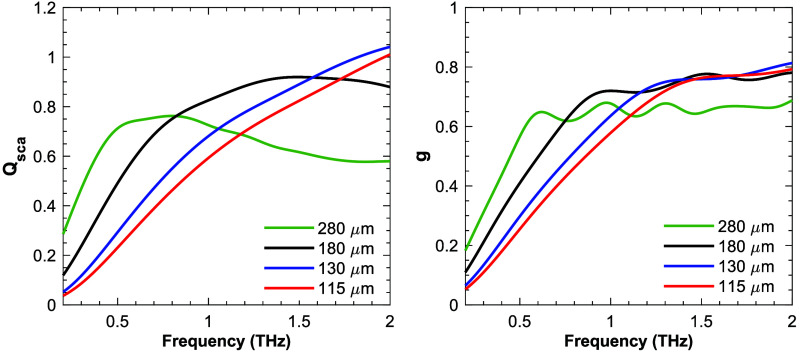
Calculated scattering efficiency and asymmetric factor for relevant particle sizes.

Using the refractive indices calculated in Sec. 2.2 and the particle sizes and volume densities determined in Sec. 3.1, the Polarized Light Monte Carlo model was used to simulate the THz polarimetric response of the final four phantoms. For each particle size, we calculated the asymmetric factor and the scattering efficiency, which is the ratio of the scattering cross-section to the geometric area of a particle. Figure 5 shows the calculated scattering efficiency and asymmetric factor for each particle size. As the particle size increases, the scattering efficiency and asymmetric factor increase in the lower frequencies up to about 1 THz.

**Fig. 6:**
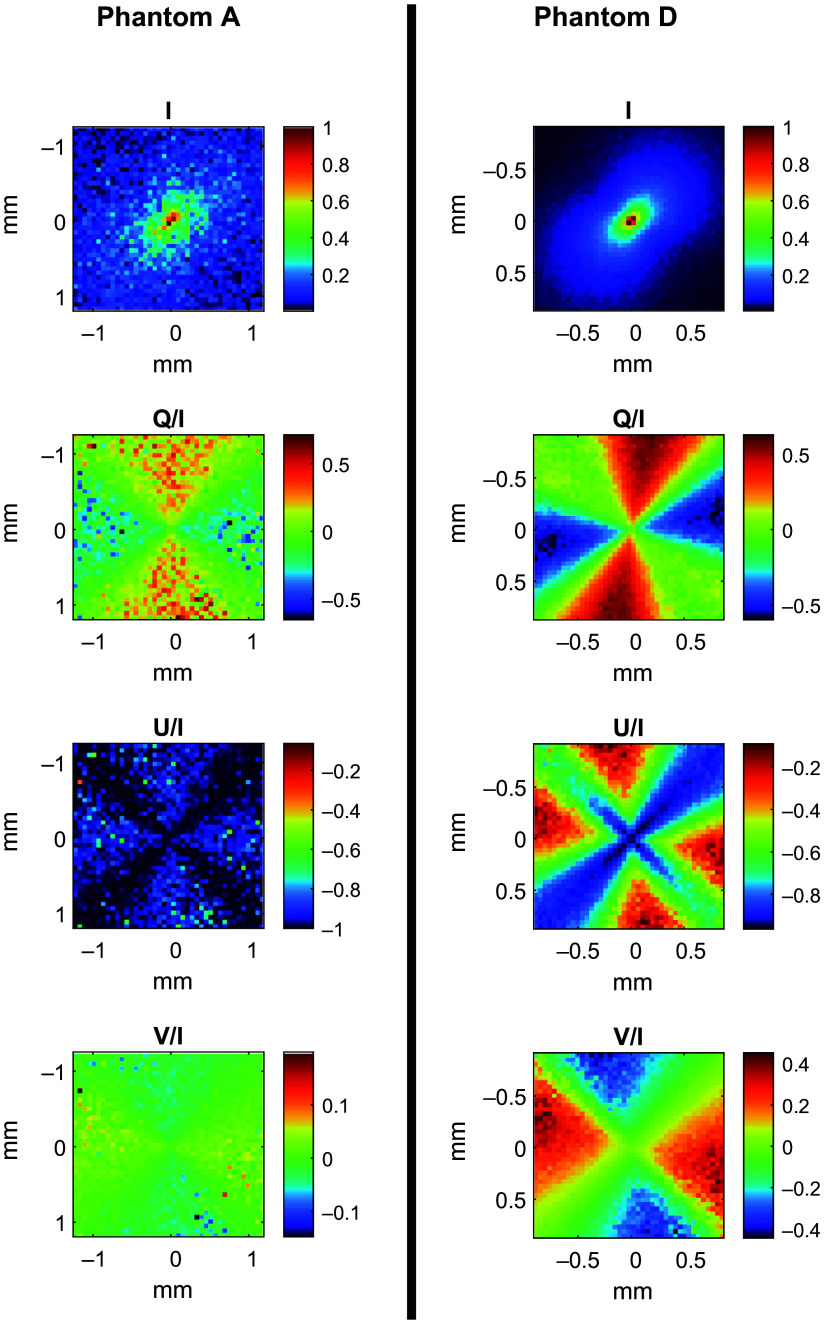
Spatial map of the simulated Stokes vector, IQUV, for phantoms A and D at 0.6 THz.

To calculate the Mueller matrix of each sample, spatial Stokes vector maps were generated for each phantom for incident light polarizations of parallel, perpendicular, and 45 deg linearly polarized, and right circularly polarized. Figure 6 presents a comparison between the Stokes parameters maps of phantoms A and D, having the smallest and largest particle sizes, respectively, when illuminated with a 45 deg linearly polarized incident 0.6 THz beam. The spatial maps reveal the greater intensity of scattered light received with the simulation of phantom D compared with phantom A, matching the expectations of a higher reflectance with a larger particle size.

**Fig. 7:**
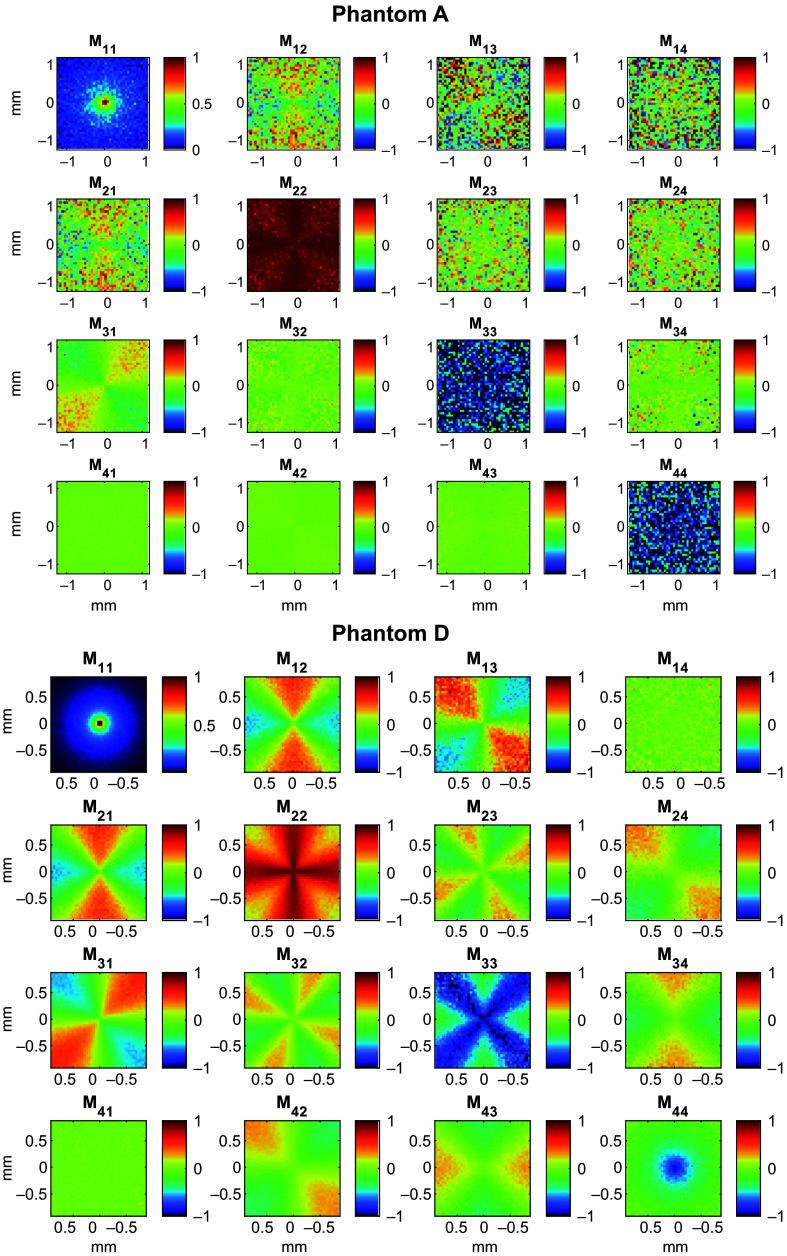
Simulated Mueller Matrix for phantoms A and D at 0.6 THz.

Using spatial maps of all four incident light polarizations, the spatial Mueller matrix maps, shown in Fig. 7, were calculated. The simulated Mueller matrices for phantoms D and A show similar angular distribution patterns, with the patterns being much less resolved with phantom A, similar to the Stokes parameter maps. The clearest difference between the two maps is present in element $M_{22}$ where the values of the angular distribution pattern of phantom D are closer to zero than the values of phantom A. This difference is also reflected in element $M_{33}$ where the values of phantom A are much closer to −1 than those of phantom D. To better analyze the differences between the simulated Mueller matrices of the phantoms, we calculated the pixel sum of each of the Mueller matrix element maps to obtain a singular Mueller matrix for each phantom. The Mueller matrices of phantoms A and D, i.e., $M_{A}$ and $M_{D}$, are given below.

$$M_{A}=3.05\times 10^{-4}[\begin{array}{cccc} 1.000 & -0.003 & 0.008 & 0.008\\ -0.003 & 0.922 & 0.004 & 0.004\\ -0.002 & -0.001 & -0.926 & 0.000\\ 0.000 & 0.000 & 0.000 & -0.850 \end{array}],$$ (7)

$$M_{D}=4.23\times 10^{-3}[\begin{array}{cccc} 1.000 & 0.000 & -0.001 & -0.001\\ 0.000 & 0.586 & 0.000 & 0.000\\ 0.000 & 0.000 & -0.586 & -0.001\\ 0.000 & 0.000 & 0.000 & -0.207 \end{array}].$$ (8)

These matrices can be decomposed using the Lu and Chipman decomposition^66^ as

$$M_{\Delta ,A}\approx [\begin{array}{cccc} 1 & 0 & 0 & 0\\ 0 & 0.921 & 0 & 0\\ 0 & 0 & 0.925 & 0\\ 0 & 0 & 0 & 0.849 \end{array}],\;M_{R,A}\approx [\begin{array}{cccc} 1 & 0 & 0 & 0\\ 0 & 1 & 0 & 0\\ 0 & 0 & -1 & 0\\ 0 & 0 & 0 & -1 \end{array}],\;M_{D,A}\approx 3.050\times 10^{-4}I,$$ (9)

$$M_{\Delta ,D}\approx [\begin{array}{cccc} 1 & 0 & 0 & 0\\ 0 & 0.586 & 0 & 0\\ 0 & 0 & 0.586 & 0\\ 0 & 0 & 0 & 0.207 \end{array}],\;M_{R,D}\approx [\begin{array}{cccc} 1 & 0 & 0 & 0\\ 0 & 1 & 0 & 0\\ 0 & 0 & -1 & 0\\ 0 & 0 & 0 & -1 \end{array}],\;M_{D,D}\approx 4.230\times 10^{-3}I,$$ (10)

where $M_{\Delta }$ is the depolarizer matrix, $M_{R}$ is the retarder matrix, and $M_{D}$ is the diattenuator matrix for phantoms A and D.

Each of the phantom’s Mueller matrices can be decomposed as approximately a uniform attenuator and a nonuniform depolarizer. Because this simulation records the reflected photons instead of transmitted photons through the medium, the diagonal value of the uniform attenuator matrix represents the diffuse reflectance of the sample. The diagonal depolarizer matrix can be represented with a single value: the degree of polarization of linear light “p”, where the depolarization matrix is diag[1, p, p, 2p-1]. Because the Mueller matrix and its decomposition can be represented in terms of only the diffuse reflectance and linear degree of polarization, only these two values are necessary to characterize the polarimetric response of these samples. These two parameters can be obtained by a measurement of either linear or circularly polarized light scattered to a diffuse angle; therefore, measurement of four different initial polarization states as would typically be done for a full Mueller matrix characterization with spherical symmetry is unnecessary in this case, according to this simulation. Using the spatial maps generated from linearly polarized light, we calculated the diffuse reflectance as the Stokes parameter I and the degree of polarization using Eq. (5) to compare with experimental results.

### 3.3 Comparison between Model and Experimental Data

For each phantom, the THz-TDS waveforms in the time domain were converted to the complex Fourier domain to produce $E_{x}$ and $E_{y}$ spectra. Stokes parameters were calculated using

$$I=\langle |E_{x}{|}^{2}\rangle +\langle |E_{y}{|}^{2}\rangle ,\;Q=\langle |E_{x}{|}^{2}\rangle -\langle |E_{y}{|}^{2}\rangle ,\;U=2R\langle E_{x}E_{y}^{*}\rangle ,\;V=-2iI\langle E_{x}E_{y}^{*}\rangle .$$ (11)

**Fig. 8:**
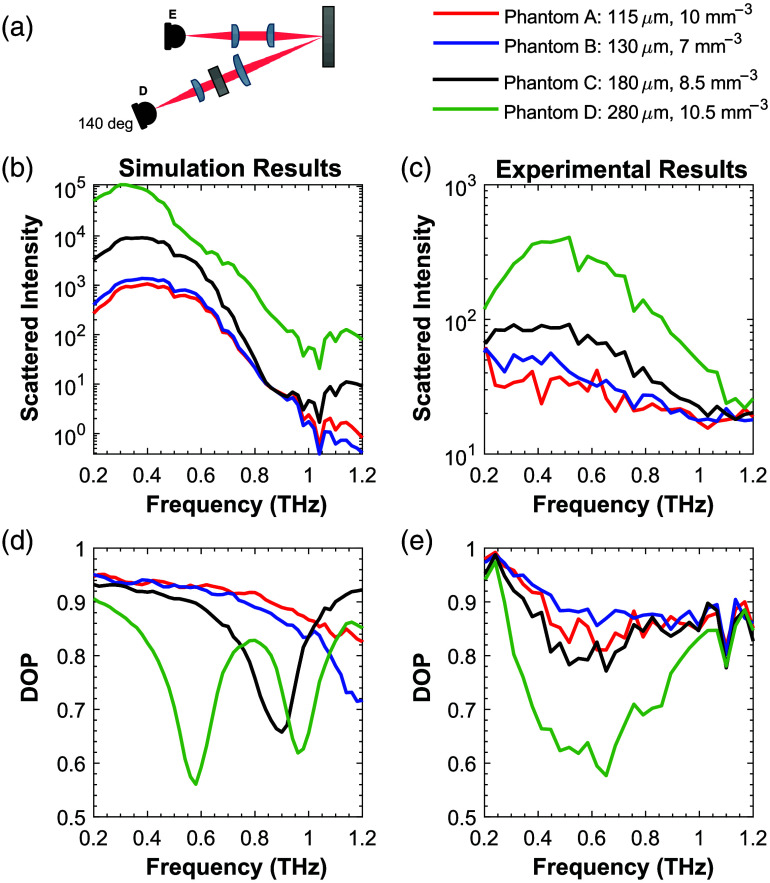
(a) Experimental setup for diffuse phantom scattering measurements. Simulated (b) and measured (c) diffuse scattered intensity from phantoms A–D are shown. Experimental results are obtained at only one detection angle, as shown in sub-figure (a). The degree of polarization (DOP) is compared between simulation (d) and experimental (e) diffuse scattered beams for different phantoms.

The intensity was taken as Stokes parameter I averaged over 100 pixels, whereas the degree of polarization (DOP) was calculated using Eq. (6) with the Stokes parameters averaged across 100 pixels with $E_{x}$ and $E_{y}$ deconvolved with an air reference taken at 0 deg (transmission). The simulated result for the diffuse reflectance was converted into reflected intensity by multiplying the simulation by the spectrum of the air reference. Figure 8 presents the simulated results for linearly polarized light alongside the experimental results for the four phantoms analyzed. The diffuse scattered intensity between 0.2 and 1.2 THz has a similar trend between the simulation and experimental results, showing a higher intensity at lower frequencies and an exponential roll-off with increasing frequency. As the diameter of the particles increases, the scattered intensity increases. The absolute values of the diffuse scattered intensity between the simulation and experiment cannot be directly compared because the simulation results represent photons from all backscattered angles, whereas the experimental data were collected only in a 20-deg detection cone. The diffuse scattered intensity is also very sensitive to slight changes in the alignment of the experimental system.

The DOP parameter, which is less susceptible to misalignments in the system, shows a trend similar to the diffuse scattered intensity results. Due to the SNR and bandwidth limitations of our system, DOP measurements beyond 0.8 THz are not as reliable.^43^^,^^44^ Figure 8 shows that with increasing frequency, the DOP of phantom D drops rapidly from 0.9, i.e., almost fully polarized light, to partially polarized with a minimum at ∼0.6  THz, which is in agreement with simulation results. In contrast, phantom C has less of a drop in DOP from 0.2 to 0.8 THz. With smaller particle sizes in phantoms A and B, a smaller frequency-dependent drop in DOP can be observed in both simulation and measured results. The simulation model suggests that to resolve the frequency minima in the DOP, which correspond to the scattering particle size, a larger bandwidth than our experimental setup would be required. Furthermore, DOP measurement is much less sensitive to a potential misalignment in the setup, so it could be a more reliable diagnostic marker than diffuse intensity.

### 3.4 Experimental Data of A Porcine Skin Burn

**Fig. 9:**
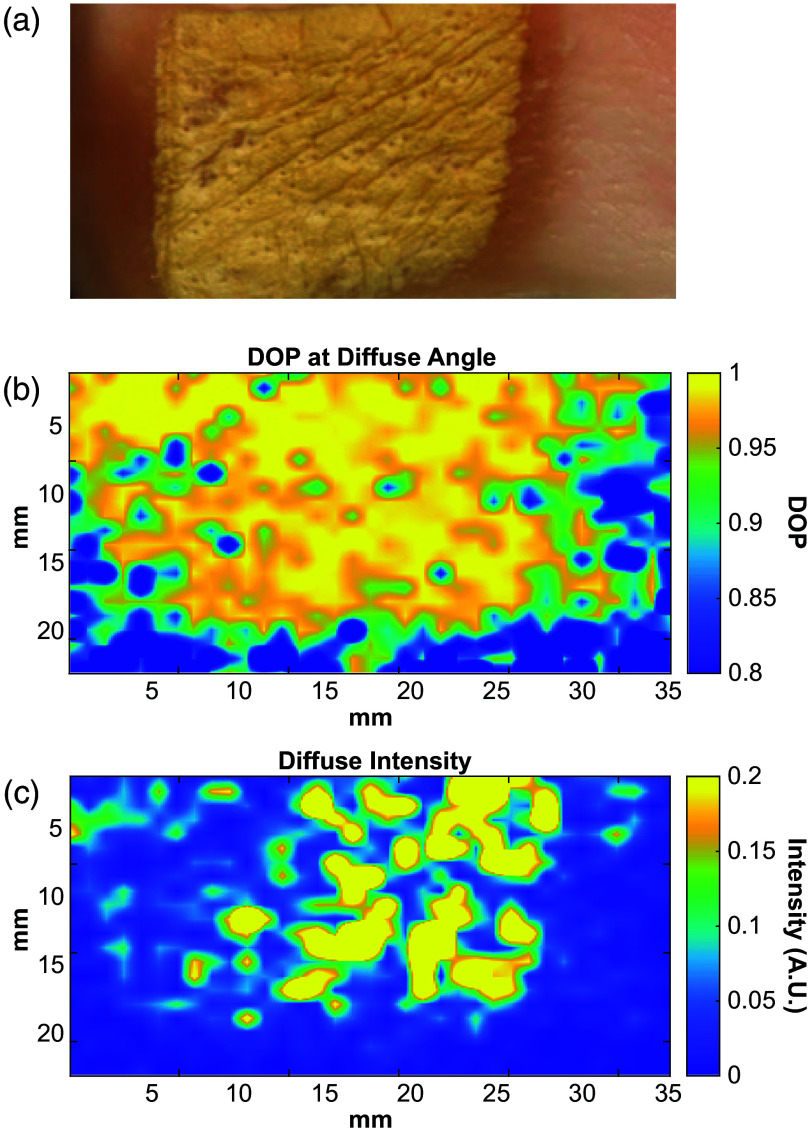
(a) Visual image of an *ex vivo* porcine skin burn scanned at a diffuse angle to produce DOP (b) and diffused scattered intensity (c) maps at 0.6 THz.

To demonstrate the potential of DOP and diffuse backscattered intensity in realistic tissue conditions, we present results of THz measurements of an ex vivo porcine skin burn. A 1-in. full-thickness burn was induced by applying a brass bar heated to 180°C to the skin for 5 min. We mounted the porcine skin in our setup at 140 deg and took measurements of a 2  cm×3.5  cm area with a 1-mm pixel size. Each pixel had 10 measurements taken with 10 time-averages. Using the 10 measurements per pixel, we calculated the intensity as the Stokes parameter I and the DOP using Eqs. (6) and (11). Figure 9 presents spatial maps of the DOP and intensity at 0.6 THz compared with a photograph of the burn area imaged. Both maps show the contrast between the burned and the healthy tissue. We have previously reported that severe burns result in an increase in the real part of the refractive index and a decrease in the absorption coefficient.^9^ This opposite effect on the complex refractive index of severely burned skin leads to an overall small change in the effective scattering cross section of tissue structures. However, the higher DOP values for the burn could be attributed to the destruction of large skin structures such as hair follicles and sweat glands, overall decreasing the average size of scatterers within the skin. The higher intensity values for the burn could be attributed to decreased absorption from the skin due to water loss from the heating of the skin in the production of the burn.

## 4 Discussion

**Fig. 10:**
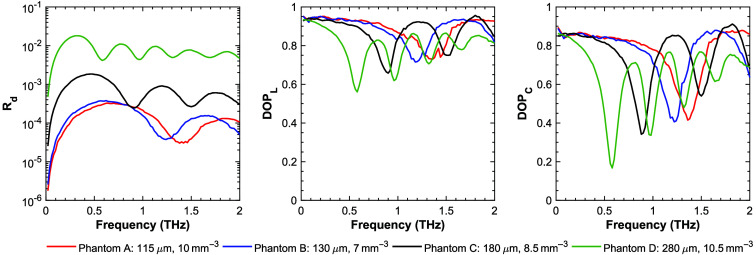
Simulated diffuse reflectance ($R_{d}$), linear DOP, and circular DOP from 0 to 2 THz.

Extending the simulation results to a larger bandwidth of 2 THz (Fig. 10) shows more clearly the frequency-dependent features of the DOP. These results reveal that as the particle size increases, the diffuse reflectance increases. In addition, the spectral location of the first minima in the DOP (for both linearly and circularly polarized light) moves to lower frequencies as the particle size increases. Xu and Arbab showed in their analysis of this simulation method that the depth of the minima in DOP curves is a function of the concentration of the particles. In other words, a larger drop in DOP corresponds to a higher concentration;^25^ therefore, spectral dependence of the DOP can allow for the determination of both the concentration and size of particles, given a frequency-dependent DOP measurement with sufficient signal-to-noise ratio and bandwidth. The circular DOP ($DOP_{C}$) is related to the linear DOP ($DOP_{L}$) by the relationship ${\mathrm{DOP}}_{C}=2{\mathrm{DOP}}_{L}-1$.^67^^,^^68^ Finally, the magnitude of the diffuse reflectance changes with both particle size and concentration. Our results suggest that the particle size-dependent pattern in the diffuse reflectance may be difficult to resolve with the current sensitivity of THz detector systems.

Given the DOP and the diffuse scattered intensity results of our phantom measurements and porcine skin burn, contrast can be seen between different sizes of scattering particles in tissue environments; however, these signal contrasts are best observed when the difference between the sizes of particles is large. Observations of smaller differences in concentration and size of scattering particles are limited by our current bandwidth and SNR. In this study, particle sizes of ∼100 to 500  μm were analyzed, corresponding to tissue features such as hair follicles, sweat glands, poorly differentiated clusters, and large tumor budding.^49^^,^^50^^,^^58^ A THz system with a larger bandwidth, such as the one developed by Xu et al. with a bandwidth of 8 THz using electro-optic crystals instead of PCA emitters and detectors,^69^ could aid in resolving these features for smaller particle size differences, such as early-stage tumor budding with a particle size of ∼10  μm.^58^ Finally, in this work, we assumed that the surface of the phantom and the *ex vivo* skin is optically flat and smooth. During the fabrication process of the phantoms, the gelatin was liquid before solidifying, allowing the surface to remain smooth. In addition, the roughness of the skin is estimated to be between 10 and 30  μm,^70^ which is on the order of λ/150−λ/10 in the bandwidth of our THz system. We have previously studied the depolarization of THz waves in backscattered speckle fields due to the surface roughness of the sample. We have reported that a significant change in the DOP value did not occur until the RMS surface height variation was greater than 60  μm.^43^ Therefore, the effect of surface roughness is not expected to be significant in the current study. In general, the DOP parameter can be spectrally affected both by surface depolarization and by scattering by the internal structures of the skin. Several signal processing and experimental measurement techniques can be used to characterize the relative contribution of each source of depolarization. Examples of these methods include wavelet signal decomposition^71^^–^^73^ and statistical speckle analysis.^43^

## 5 Conclusion

We have presented simulation and experimental results for Mie scattering of THz light due to structures in biological tissue, such as hair follicles, sweat glands, and tumor budding. Simulations revealed the potential for diffuse backscattered intensity and the degree of polarization (DOP) parameter to distinguish between different scattering scenarios. Simulated Mueller matrices showed that for spherical particles in an absorbing medium, only one polarization measurement from either linearly or circularly polarized light is needed to construct a full Mueller matrix of the sample. In addition, we decomposed the Mueller matrices of the tissue according to the Lu-Chipman formalism and summarized the key sources of the scattering signal contrast in highly absorptive media. Experimental measurements of phantoms of moderately sized tumor budding and poorly differentiated clusters confirmed the frequency-dependent patterns from our simulation and showed the potential of DOP and diffuse scattered intensity for diagnosis. Finally, experimental measurements of a porcine skin burn showed contrast between burned regions and healthy regions of tissue, demonstrating the ability of this technique to distinguish between disease states in *ex vivo* tissue. Future work in this direction would include extending our phantom measurements to *ex vivo* cancer tissue, increasing the bandwidth of the measurements to better resolve the frequency-dependent features.

## Data Availability

Datasets and analysis codes for this article are available upon request to Dr. Hassan Arbab, hassan.arbab@stonybrook.edu.

## References

[1] Leitenstorfer A. et al., “The 2023 terahertz science and technology roadmap,” J. Phys. D: Appl. Phys. 56(22), 223001 (2023). JPAPBE 0022-3727 10.1088/1361-6463/acbe4c

[2] Chen X. et al., “Terahertz (THz) biophotonics technology: instrumentation, techniques, and biomedical applications,” Chem. Phys. Rev. 3(1), 011311 (2022). 10.1063/5.0068979

[3] Ashworth P. C. et al., “Terahertz pulsed spectroscopy of freshly excised human breast cancer,” Opt. Express 17, 12444–12454 (2009). OPEXFF 1094-4087 10.1364/OE.17.012444 19654646

[r4] El-Shenawee M. et al., “Cancer detection in excised breast tumors using terahertz imaging and spectroscopy,” Biomed. Spectrosc. Imaging 8, 1–9 (2019). 10.3233/BSI-190187 32566474 PMC7304303

[r5] Fan B. Neel V. A. Yaroslavsky A. N., “Multimodal imaging for nonmelanoma skin cancer margin delineation,” Lasers Surg. Med. 49(3), 319–326 (2017). LSMEDI 0196-8092 10.1002/lsm.22552 27490843

[6] Sim Y. C. et al., “Terahertz imaging of excised oral cancer at frozen temperature,” Biomed. Opt. Express 4, 1413–1421 (2013). BOEICL 2156-7085 10.1364/BOE.4.001413 24010003 PMC3756582

[7] Khani M. E. et al., “Accurate and early prediction of the wound healing outcome of burn injuries using the wavelet Shannon entropy of terahertz time-domain waveforms,” J. Biomed. Opt. 27, 116001 (2022). JBOPFO 1083-3668 10.1117/1.JBO.27.11.116001 36348509 PMC9641274

[r8] Osman O. B. et al., “Deep neural network classification of in vivo burn injuries with different etiologies using terahertz time-domain spectral imaging,” Biomed. Opt. Express 13, 1855–1868 (2022). BOEICL 2156-7085 10.1364/BOE.452257 35519269 PMC9045889

[9] Khani M. E. et al., “Triage of in vivo burn injuries and prediction of wound healing outcome using neural networks and modeling of the terahertz permittivity based on the double Debye dielectric parameters,” Biomed. Opt. Express 14, 918–931 (2023). BOEICL 2156-7085 10.1364/BOE.479567 36874480 PMC9979665

[10] Chen A. et al., “Assessing corneal endothelial damage using terahertz time-domain spectroscopy and support vector machines,” Sensors 22, 9071 (2022). SNSRES 0746-9462 10.3390/s22239071 36501773 PMC9735956

[r11] Gavdush A. A. et al., “Terahertz dielectric spectroscopy of human brain gliomas and intact tissues ex vivo: double-Debye and double-overdamped-oscillator models of dielectric response,” Biomed. Opt. Express 12, 69–83 (2021). BOEICL 2156-7085 10.1364/BOE.411025 33659071 PMC7899500

[r12] Taylor Z. D. et al., “THz and mm-wave sensing of corneal tissue water content: in vivo sensing and imaging results,” IEEE Trans. THz. Sci. Technol. 5, 184–196 (2015). 10.1109/TTHZ.2015.2392628 PMC4493917 26161292

[13] Borovkova M. et al., “Terahertz time-domain spectroscopy for non-invasive assessment of water content in biological samples,” Biomed. Opt. Express 9, 2266–2276 (2018). BOEICL 2156-7085 10.1364/BOE.9.002266 29760985 PMC5946786

[14] Pickwell E. et al., “In vivo study of human skin using pulsed terahertz radiation,” Phys. Med. Biol. 49, 1595 (2004). PHMBA7 0031-9155 10.1088/0031-9155/49/9/001 15152918

[r15] Bennett D. B. et al., “Stratified media model for terahertz reflectometry of the skin,” IEEE Sens. J. 11, 1253–1262 (2011). ISJEAZ 1530-437X 10.1109/JSEN.2010.2088387

[16] Zaytsev K. I. et al., “Highly accurate in vivo terahertz spectroscopy of healthy skin: variation of refractive index and absorption coefficient along the human body,” IEEE Trans. THz. Sci. Technol. 5, 817–827 (2015). 10.1109/TTHZ.2015.2460677

[17] Lugli A. et al., “Recommendations for reporting tumor budding in colorectal cancer based on the International Tumor Budding Consensus Conference (ITBCC) 2016,” Mod. Pathol. 30, 1299–1311 (2017). MODPEO 0893-3952 10.1038/modpathol.2017.46 28548122

[18] Venkatesh D. Smitha T., “Cell budding,” J. Oral Maxillofacial Pathol. 23(3), 330–332 (2019). 10.4103/jomfp.JOMFP_309_19 PMC6948054 31942109

[19] Reggiani Bonetti L. et al., “Poorly differentiated clusters (PDC) in colorectal cancer: what is and ought to be known,” Diagn. Pathol. 11, 31 (2016). DMPAES 1052-9551 10.1186/s13000-016-0481-7 27004798 PMC4802878

[20] Lugli A. et al., “Tumour budding in solid cancers,” Nat. Rev. Clin. Oncol. 18(2), 101–115 (2021). 10.1038/s41571-020-0422-y 32901132

[21] Arbab M. H. et al., “Terahertz reflectometry of burn wounds in a rat model,” Biomed. Opt. Express 2, 2339–2347 (2011). BOEICL 2156-7085 10.1364/BOE.2.002339 21833370 PMC3149531

[22] Arbab M. H. et al., “Terahertz spectroscopy for the assessment of burn injuries in vivo,” J. Biomed. Opt. 18, 077004 (2013). JBOPFO 1083-3668 10.1117/1.JBO.18.7.077004 23860943

[23] Ghosh N. Vitkin A. I., “Tissue polarimetry: concepts, challenges, applications, and outlook,” J. Biomed. Opt. 16, 110801 (2011). JBOPFO 1083-3668 10.1117/1.3652896 22112102

[24] Hielscher A. H. et al., “Diffuse backscattering Mueller matrices of highly scattering media,” Opt. Express 1, 441–453 (1997). OPEXFF 1094-4087 10.1364/OE.1.000441 19377568

[25] Xu K. Arbab M. H., “Terahertz polarimetric imaging of biological tissue: Monte Carlo modeling of signal contrast mechanisms due to Mie scattering,” Biomed. Opt. Express 15, 2328–2342 (2024). BOEICL 2156-7085 10.1364/BOE.515623 38633080 PMC11019684

[26] Garcia-Uribe A. et al., “In vivo diagnosis of melanoma and nonmelanoma skin cancer using oblique incidence diffuse reflectance spectrometry,” Cancer Res. 72(11), 2738–2745 (2012). CNREA8 0008-5472 10.1158/0008-5472.CAN-11-4027 22491533 PMC3367032

[27] Nishizawa N. Kuchimaru T., “Depth estimation of tumor invasion in early gastric cancer using scattering of circularly polarized light: Monte Carlo Simulation study,” J. Biophotonics 15(10), e202200062 (2022). 10.1002/jbio.202200062 35666013

[28] Altoe M. L. et al., “Changes in diffuse optical tomography images during early stages of neoadjuvant chemotherapy correlate with tumor response in different breast cancer subtypes,” Clin. Cancer Res. 27(7), 1949–1957 (2021). 10.1158/1078-0432.CCR-20-1108 33451976 PMC8128376

[29] Keller M. D. et al., “Autofluorescence and diffuse reflectance spectroscopy and spectral imaging for breast surgical margin analysis,” Lasers Surg. Med. 42(1), 15–23 (2010). LSMEDI 0196-8092 10.1002/lsm.20865 20077490

[30] A’Amar O. M. et al., “Comparison of elastic scattering spectroscopy with histology in ex vivo prostate glands: potential application for optically guided biopsy and directed treatment,” Lasers Med. Sci. 28(5), 1323–1329 (2013). 10.1007/s10103-012-1245-6 23247663

[31] Antonelli M.-R. et al., “Mueller matrix imaging of human colon tissue for cancer diagnostics: how Monte Carlo modeling can help in the interpretation of experimental data,” Opt. Express 18, 10200–10208 (2010). OPEXFF 1094-4087 10.1364/OE.18.010200 20588874

[32] Pierangelo A. et al., “Ex-vivo characterization of human colon cancer by Mueller polarimetric imaging,” Opt. Express 19, 1582–1593 (2011). OPEXFF 1094-4087 10.1364/OE.19.001582 21263698

[33] Arifler D. et al., “Spatially resolved reflectance spectroscopy for diagnosis of cervical precancer: Monte Carlo modeling and comparison to clinical measurements,” J. Biomed. Opt. 11, 064027 (2006). JBOPFO 1083-3668 10.1117/1.2398932 17212550

[34] Steelman Z. A. et al., “Light-scattering methods for tissue diagnosis,” Optica 6(4), 479–489 (2019). 10.1364/OPTICA.6.000479 33043100 PMC7544148

[35] Fu Y. et al., “Flexible 3x3 Mueller matrix endoscope prototype for cancer detection,” IEEE Trans. Instrum. Meas. 67, 1700–1712 (2018). IEIMAO 0018-9456 10.1109/TIM.2018.2803847

[36] Liu T. et al., “Distinguishing structural features between Crohn’s disease and gastrointestinal luminal tuberculosis using Mueller matrix derived parameters,” J. Biophotonics 12(12), e201900151 (2019). 10.1002/jbio.201900151 31465142

[37] Rodriguez-Diaz E. et al., “Endoscopic histological assessment of colonic polyps by using elastic scattering spectroscopy,” Gastrointest. Endosc. 81(3), 539–547 (2015). 10.1016/j.gie.2014.07.012 25257128 PMC5533077

[38] Trout R. M. et al., “Polarization enhanced laparoscope for improved visualization of tissue structural changes associated with peritoneal cancer metastasis,” Biomed. Opt. Express 13, 571 (2022). BOEICL 2156-7085 10.1364/BOE.443926 35284190 PMC8884200

[39] Doradla P. et al., “Detection of colon cancer by continuous-wave terahertz polarization imaging technique,” J. Biomed. Opt. 18, 090504 (2013). JBOPFO 1083-3668 10.1117/1.JBO.18.9.090504

[40] Gurjar N. Bailey K. El-Shenawee M. O., “Polarimetry terahertz imaging of human breast cancer surgical specimens,” J. Med. Imaging 11, 065503 (2024). JMEIET 0920-5497 10.1117/1.JMI.11.6.065503 PMC11619717 39649775

[41] Lopushenko I. et al., “Exploring the evolution of circular polarized light backscattered from turbid tissue-like disperse medium utilizing generalized Monte Carlo modeling approach with a combined use of Jones and Stokes-Mueller formalisms,” J. Biomed. Opt. 29, 052913 (2023). JBOPFO 1083-3668 10.1117/1.JBO.29.5.052913 38089555 PMC10715447

[42] Chen X. et al., “Robust and accurate terahertz time-domain spectroscopic ellipsometry,” Photonics Res. 6, 768–775 (2018). 10.1364/PRJ.6.000768

[r43] Xu K. Harris Z. B. Arbab M. H., “Polarimetric imaging of back-scattered terahertz speckle fields using a portable scanner,” Opt. Express 31, 11308–11319 (2023). OPEXFF 1094-4087 10.1364/OE.482733 37155769 PMC10316681

[r44] Harris Z. B. Xu K. Arbab M. H., “A handheld polarimetric imaging device and calibration technique for accurate mapping of terahertz stokes vectors,” Sci. Rep. 14, 17714 (2024). SRCEC3 2045-2322 10.1038/s41598-024-68530-4 39085453 PMC11292021

[r45] Castro-Camus E. et al., “Polarization-sensitive terahertz detection by multicontact photoconductive receivers,” Appl. Phys. Lett. 86(25), 254102 (2005). APPLAB 0003-6951 10.1063/1.1951051

[r46] Morris C. M. et al., “Polarization modulation time-domain terahertz polarimetry,” Opt. Express 20(11), 12303–12317 (2012). OPEXFF 1094-4087 10.1364/OE.20.012303 22714218

[47] Bulgarevich D. S. et al., “A polarization-sensitive 4-contact detector for terahertz time-domain spectroscopy,” Opt. Express 22(9), 10332–10340 (2014). OPEXFF 1094-4087 10.1364/OE.22.010332 24921735

[48] Khani M. E. Osman O. B. Arbab M. H., “Diffuse terahertz spectroscopy in turbid media using a wavelet-based bimodality spectral analysis,” Sci. Rep. 11, 22804 (2021). SRCEC3 2045-2322 10.1038/s41598-021-02068-7 34815438 PMC8611087

[49] Chopra N. et al., “THz time-domain spectroscopy of human skin tissue for in-body nanonetworks,” IEEE Trans. THz. Sci. Technol. 6, 803–809 (2016). 10.1109/TTHZ.2016.2599075

[50] Tripathi S. R. et al., “Morphology of human sweat ducts observed by optical coherence tomography and their frequency of resonance in the terahertz frequency region,” Sci. Rep. 5, 9071 (2015). SRCEC3 2045-2322 10.1038/srep09071 25766116 PMC4357862

[51] Nourinovin S. et al., “Terahertz characterization of ordinary and aggressive types of oral squamous cell carcinoma as a function of cancer stage and treatment efficiency,” IEEE Trans. Instrum. Meas. 72, 1–9 (2023). IEIMAO 0018-9456 10.1109/TIM.2023.331246937323850

[52] Ji Y. B. et al., “Investigation of keratinizing squamous cell carcinoma of the tongue using terahertz reflection imaging,” J. Infrared Millim. THz. Waves 40(2), 247–256 (2019). 10.1007/s10762-018-0562-7

[53] Kucheryavenko A. et al., “Terahertz-wave scattering in tissues: examining the limits of the applicability of effective-medium theory,” Phys. Rev. Appl. 20, 054050 (2023). PRAHB2 2331-7019 10.1103/PhysRevApplied.20.054050

[r54] Saxena S. et al., “Limitations of effective medium models for tissue phantoms in the THz frequency range,” Sci. Rep. 14, 22968 (2024). SRCEC3 2045-2322 10.1038/s41598-024-70590-5 39362921 PMC11450206

[r55] Tamminen A. et al., “Extraction of thickness and water-content gradients in hydrogel-based water-backed corneal phantoms via submillimeter-wave reflectometry,” IEEE Trans. THz. Sci. Technol. 11, 647–659 (2021). 10.1109/TTHZ.2021.3099058

[56] Tamminen A. et al., “Submillimeter-wave permittivity measurements of bound water in collagen hydrogels via frequency domain spectroscopy,” IEEE Trans. THz. Sci. Technol. 11, 538–547 (2021). 10.1109/TTHZ.2021.3088273

[57] Schneider C. A. Rasband W. S. Eliceiri K. W., “NIH image to ImageJ: 25 years of image analysis,” Nat. Methods 9(7), 671–675 (2012).1548-7091 10.1038/nmeth.2089 22930834 PMC5554542

[58] Boxberg M. et al., “Tumour budding activity and cell nest size determine patient outcome in oral squamous cell carcinoma: proposal for an adjusted grading system,” Histopathology 70(7), 1125–1137 (2017). HISTDD 1365-2559 10.1111/his.13173 28122134

[59] Harris Z. B. Arbab M. H., “Terahertz phasr scanner with 2 kHz, 100 ps time-domain trace acquisition rate and an extended field-of-view based on a heliostat design,” IEEE Trans. THz. Sci. Technol. 12, 619–632 (2022). 10.1109/TTHZ.2022.3200210 PMC9757810 36531441

[60] Chang T. et al., “Terahertz dielectric spectroscopy based thermal aging analysis of polypropylene,” IEEE Trans. THz. Sci. Technol. 10, 363–369 (2020). 10.1109/TTHZ.2020.2992218

[61] Jin Y.-S. Kim G.-J. Jeon S.-G., “Terahertz dielectric properties of polymers,” J. Kor. Phys. Soc. 49, 513–517 (2006). KPSJAS 0374-4884

[62] Ney M. Abdulhalim I., “Ultrahigh polarimetric image contrast enhancement for skin cancer diagnosis using InN plasmonic nanoparticles in the terahertz range,” J. Biomed. Opt. 20, 125007 (2015). JBOPFO 1083-3668 10.1117/1.JBO.20.12.125007 26720872

[63] Feldman Y. et al., “The electromagnetic response of human skin in the millimetre and submillimetre wave range,” Phys. Med. Biol. 54, 3341 (2009). PHMBA7 0031-9155 10.1088/0031-9155/54/11/005 19430110

[64] Ramella-Roman J. C. Prahl S. A. Jacques S. L., “Three Monte Carlo programs of polarized light transport into scattering media: part I,” Opt. Express 13, 4420–4438 (2005). OPEXFF 1094-4087 10.1364/OPEX.13.004420 19495358

[65] Yang P. et al., “Inherent and apparent scattering properties of coated or uncoated spheres embedded in an absorbing host medium,” Appl. Opt. 41, 2740–2759 (2002). APOPAI 0003-6935 10.1364/AO.41.002740 12027161

[66] Lu S.-Y. Chipman R. A., “Interpretation of Mueller matrices based on polar decomposition,” J. Opt. Soc. Amer. A 13, 1106–1113 (1996). JOAOD6 0740-3232 10.1364/JOSAA.13.001106

[67] Mishchenko M. I. Hovenier J. W., “Depolarization of light backscattered by randomly oriented nonspherical particles,” Opt. Lett. 20, 1356–1358 (1995). OPLEDP 0146-9592 10.1364/OL.20.001356 19862013

[68] van de Hulst H. C., Light Scattering by Small Particles, Dover Publications (1981).

[69] Xu K. Liu M. Arbab M. H., “Broadband terahertz time-domain polarimetry based on air plasma filament emissions and spinning electro-optic sampling in GaP,” Appl. Phys. Lett. 120, 181107 (2022). APPLAB 0003-6951 10.1063/5.0087127 35539361 PMC9068238

[70] Maiti R. et al., “In vivo measurement of skin surface strain and sub-surface layer deformation induced by natural tissue stretching,” J. Mech. Behav. Biomed. Mater. 62, 556–569 (2016). 10.1016/j.jmbbm.2016.05.035 27310571

[71] Arbab M. H. et al., “Effect of surface scattering on terahertz time domain spectroscopy of chemicals,” Proc. SPIE 6893, 68930C (2008). PSISDG 0277-786X 10.1117/12.769015

[r72] Arbab M. H. et al., “Application of wavelet transforms in terahertz spectroscopy of rough surface targets,” Proc. SPIE 7601, 760106 (2010). PSISDG 0277-786X 10.1117/12.845944

[73] Khani M. E. Arbab M. H., “Translation-invariant zero-phase wavelet methods for feature extraction in terahertz time-domain spectroscopy,” Sensors 22(6), 2305 (2022). SNSRES 0746-9462 10.3390/s22062305 35336476 PMC8952727

